# Acceptability, Continuation, and User Experience of Menstrual Cup Usage Among Married Women: A Prospective Observational Study

**DOI:** 10.7759/cureus.104361

**Published:** 2026-02-27

**Authors:** T Naga Sai Keerthi, Shobha Shiragur, S R Bidri, Shreedevi Kori, Preeti Malapure, Santosh Arakeri

**Affiliations:** 1 Obstetrics and Gynaecology, Shri B. M. Patil Medical College, Hospital and Research Centre, BLDE (Deemed to be University), Vijayapura, IND

**Keywords:** acceptability, menstrual cup, menstrual hygiene management, reproductive health, sustainable menstruation

## Abstract

Introduction: Menstrual hygiene management is an essential component of women’s reproductive health, influencing physical well-being, dignity, and quality of life. Despite the availability of disposable menstrual products, concerns related to environmental burden, cost, and health risks persist. Menstrual cups offer a reusable, potentially safe, and eco-friendly alternative; however, their acceptability and continued use remain limited in many settings. The present study was undertaken to evaluate the acceptability, continuation, and user experience of menstrual cup usage among married women of reproductive age.

Materials and methods: This prospective observational study was conducted from March 2024 to September 2025 at a tertiary care teaching hospital in Vijayapura, Karnataka on 142 healthy married women of reproductive age group. Baseline data on sociodemographic characteristics, obstetric and menstrual history, menstrual hygiene practices, and awareness of menstrual cups were collected using a structured proforma. Eligible participants received individual standardized counselling with demonstration of insertion, removal, and cleaning techniques. Follow-up assessments were conducted at one, three, and six months to evaluate acceptability, continuation of use, symptom profile, and utility. Data were analyzed using IBM SPSS Statistics for Windows, Version 26 (Released 2018; IBM Corp., Armonk, New York, United States), with categorical variables expressed as frequencies and percentages and statistical significance set at p < 0.05.

Results: Most participants were aged 26-35 years (100 (70.4%)). Awareness regarding menstrual cup use was present in 87 (61.3%), while willingness to use was high at 122 (85.9%). Continued use showed a gradual decline across follow-up, with 94 (66.2%) of participants continuing menstrual cup use at six months. Symptoms such as pain, discomfort, vaginal irritation, discharge, and leakage were more common during early use but reduced significantly over subsequent follow-ups (p < 0.001). User-reported ease of wearing, removal, and cleaning improved with continued use.

Conclusion: Menstrual cups showed good acceptability and progressive user adaptation over time, though not all participants continued long term. While no serious adverse events were reported, larger controlled studies are required to formally establish their long-term safety and comparative effectiveness. With appropriate counselling and follow-up support, menstrual cups represent a practical and sustainable alternative for menstrual hygiene management.

## Introduction

Menstruation is a natural and physiological process occurring from puberty to menopause and represents an essential indicator of women’s reproductive health [1]. It involves the cyclical shedding of the endometrial lining, accompanied by blood and tissue [1]. The World Health Organization recognizes menstruation not merely as a hygienic issue but as an integral component of physical, emotional, and social well-being, emphasizing the need for safe, dignified, and hygienic menstrual management [2]. Menstrual hygiene management encompasses the use of clean absorbent or collection materials, access to water and sanitation facilities, privacy for changing menstrual products, and safe disposal mechanisms [3].

Globally, approximately 1.9 billion women, nearly one-fourth of the world’s population, are of menstruating age, spending an estimated 65 days annually managing menstrual flow [4,5]. On any given day, nearly 500 million lack access to appropriate menstrual hygiene products and facilities [6]. This challenge is particularly pronounced in low- and middle-income countries, where inadequate water, sanitation, and hygiene infrastructure, limited awareness, social stigma, and poor disposal systems contribute to health risks, discomfort, and compromised quality of life [7]. Inadequate menstrual fluid management often leads to leakage, infections, irritation, and reduced participation in daily activities [8].

Conventional menstrual hygiene products, such as disposable sanitary pads and tampons, while widely used, pose environmental and health concerns [9]. In India, menstrual hygiene products contribute significantly to non-biodegradable waste, with an estimated 11,000 products discarded by a woman over her lifetime, taking hundreds of years to decompose [10]. Additionally, chemical constituents such as phthalates, parabens, and bisphenols present in some disposable products have been implicated in altering vaginal flora and increasing the risk of irritation and infections [11]. Alternatives such as reusable cloth pads and biodegradable pads have limitations related to maintenance, drying requirements, cost, and accessibility [12].

Menstrual cups, made of medical-grade silicone, represent a reusable, non-toxic, cost-effective, and environmentally sustainable alternative [13]. One menstrual cup can replace hundreds of disposable pads or tampons over several years [13]. Despite growing awareness and evidence from studies in India and other countries demonstrating high acceptability and user satisfaction, robust long-term comparative evidence remains limited, and actual usage remains low, even among educated and urban women [14,15]. Cultural taboos, fear of insertion and removal, concerns regarding leakage, and lack of peer exposure contribute to the gap between awareness and practice [16]. Moreover, there is limited community-based longitudinal data assessing acceptability and continued use of menstrual cups among married women from the general population. Hence, the present study was conducted to evaluate the acceptability and consistency of menstrual cup usage among married women of reproductive age.

## Materials and methods

Study design

This was a prospective observational cohort study conducted from March 2024 to September 2025 at B.L.D.E. (Deemed to be University), Shri B.M. Patil Medical College Hospital and Research Centre, Vijayapura, in the Department of Obstetrics and Gynecology. Participant enrolment was carried out through health camps, institutional outreach activities, and outpatient recruitment within the Vijayapura locality. The study protocol was approved by the Institutional Ethics Committee (IEC No.: BLDE(DU)/IEC-SBMPMC/062/2023-24).

Inclusion and exclusion criteria

All married women in the reproductive age group were eligible for inclusion. Women aged below 18 years, those with menorrhagia, endometriosis, uterine fibroids, active genital infections, existing intrauterine contraceptive device use, or known allergy to silicone were excluded from the study to avoid confounding of menstrual symptoms and to ensure appropriate and supervised initiation of menstrual cup usage.

Sample size

Sample size calculation was based on a previous study by Gharacheh et al. [17], which reported a prevalence of vaginal irritation of 9.3% among menstrual cup users. With a confidence level of 96%, a margin of error of 5%, and a significance level of 4%, the required sample size was calculated using the formula: n = (Z² × p × (1 − p)) / d², where Z = 2.05, p = 0.093, and d = 0.05. The minimum required sample size was 142 participants.

Participant recruitment and flow

A total of 250 women of reproductive age were approached and counselled regarding menstrual cup use. Of these, 82 women declined participation after counselling due to personal, cultural, or family-related reasons, and 26 women were excluded based on predefined exclusion criteria. Finally, 142 eligible women who provided written informed consent were enrolled and constituted the study cohort. All enrolled participants were successfully contacted at each follow-up point. Discontinuation reflected active refusal or cessation of menstrual cup use rather than loss to follow-up.

Informed consent and counselling procedure

Written informed consent was obtained after eligibility screening and before enrolment into the study. All enrolled participants received individual, face-to-face standardized counselling by trained healthcare personnel. Counselling included education on benefits of menstrual cups, step-by-step demonstration of insertion and removal using pelvic models, instructions on cleaning and sterilization, and clarification of myths and concerns related to usage. Each counselling session lasted approximately 15-20 minutes.

Data collection and baseline assessment

Baseline data were collected using a structured proforma (see the Appendices) capturing sociodemographic characteristics, marital and obstetric history, menstrual characteristics, menstrual hygiene practices, and prior awareness regarding menstrual cup usage.

Follow-up methodology

**Figure 1:**
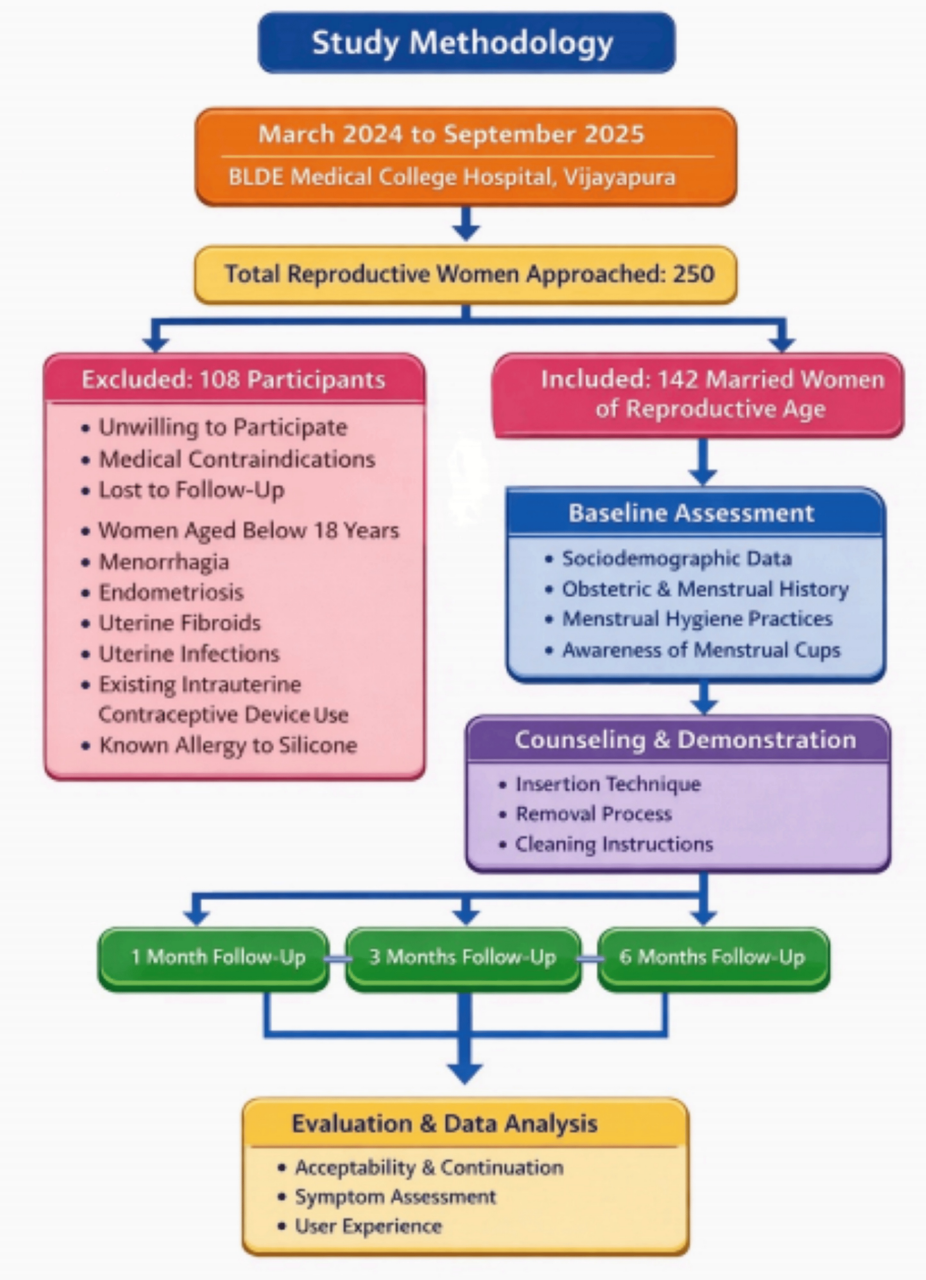
Study flow chart

Participants were followed up at one month, three months, and six months after initiation of menstrual cup use. Follow-up assessments were conducted through outpatient visits or telephonic interviews depending on participant convenience. Participants who missed scheduled visits were contacted telephonically to minimize loss to follow-up. Continuation or discontinuation of menstrual cup use, reasons for discontinuation, adverse events, and user experience were documented at each follow-up. Symptoms (pain, lower abdominal pain, vaginal discharge, irritation, discomfort, and leakage) were recorded as binary self-reported responses (present/absent) without the use of a validated severity scale. The number lost to follow-up and reasons for dropout were recorded (Figure 1).

Outcome measures and definition of effectiveness

The primary outcome was the continuation rate of menstrual cup usage at six months. Secondary outcomes included user-reported symptom profile, ease of insertion and removal, comfort during wear, leakage protection, and overall satisfaction across follow-up visits. Given the single-arm observational design, no comparator group was included, and outcomes reflect longitudinal user-reported adaptation rather than comparative effectiveness.

Statistical analysis

Data were entered into Microsoft Excel and analyzed using IBM SPSS Statistics for Windows, Version 26 (Released 2018; IBM Corp., Armonk, New York, United States). Categorical variables were expressed as frequencies and percentages and analyzed using the Chi-square test. A p-value of <0.05 was considered statistically significant.

## Results

Most participants were aged 26-30 years (52 (36.6%)) and 31-35 years (48 (33.8%)), followed by 36-40 years (36 (25.4%)), with only six participants (4.2%) in the 20-25-year group. Sanitation workers constituted the largest occupational group (36 (25.4%)), followed by doctors (28 (19.7%)), staff nurses (24 (16.9%)), and homemakers (23 (16.2%)). Clerks accounted for 9 (6.3%), teachers 8 (5.6%), and both laboratory technicians and maids 6 (4.2%) each, while daily wage laborers were least represented (2 (1.4%)). The majority belonged to the upper middle socioeconomic class (83 (58.5%)), followed by the upper class (51 (35.9%)), with only five participants (3.5%) in the upper lower class and three participants (2.1%) in the lower middle class (Table 1).

**Table 1:** Sociodemographic profile of study participants (n = 142) Distribution of participants according to age group, occupation, and socioeconomic status. Values are presented as number and percentage of the total study population.

Variable	Category	Number (n)	Percentage (%)
Age (years)	20–25	6	4.2
	26–30	52	36.6
	31–35	48	33.8
	36–40	36	25.4
Occupation	Sanitation worker	36	25.4
	Doctor	28	19.7
	Staff nurse	24	16.9
	Homemaker	23	16.2
	Clerk	9	6.3
	Teacher	8	5.6
	Lab technician	6	4.2
	Maid	6	4.2
	Daily wage labour	2	1.4
Socioeconomic status	Upper class	51	35.9
	Upper middle class	83	58.5
	Upper lower class	5	3.5
	Lower middle class	3	2.1

Most women had a marital duration of 11-20 years (90 (63.1%)), while 49 (34.8%) had been married for 1-10 years and 3 (2.1%) for 21-30 years. Multiparity was common, with parity two (40 (28.2%)) and parity three (43 (30.3%)) forming the largest groups, whereas nulliparous women accounted for 25 (17.6%) and parity one for 21 (14.8%). Higher parity (≥4) was observed in 13 (9.1%) participants. A history of lower segment cesarean section (LSCS) was present in 40 (28.2%) women, while 102 (71.8%) had no previous LSCS (Table 2).

**Table 2:** Marital and obstetric characteristics (n = 142) Distribution of participants based on the marital duration, parity, and history of lower segment cesarean section (LSCS). Data are shown as number and percentage.

Variable	Category	Number (n)	Percentage (%)
Marital duration (years)	1–10	49	34.8
	11–20	90	63.1
	21–30	3	2.1
Parity	0	25	17.6
	1	21	14.8
	2	40	28.2
	3	43	30.3
	4	9	6.3
	5	4	2.8
History of LSCS	Present	40	28.2
	Absent	102	71.8

Menarche occurred at 13-14 years in most participants (113 (79.6%)), followed by ≤12 years in 20 participants (14.1%) and ≥15 years in nine participants (6.3%). Regular menstrual cycles were reported by 116 (82.0%), whereas 26 (18.0%) had irregular cycles. The duration of menstrual flow was 3-4 days in 58 (40.8%) and 4-5 days in 61 (43.0%), while 23 (16.2%) reported flow ≥5 days. Dysmenorrhea was reported by 12 (8.5%) women and passage of clots by seven (4.9%) women, indicating low menstrual morbidity (Table 3).

**Table 3:** Menstrual characteristics of study participants (n = 142) Menstrual profile including age at menarche, cycle regularity, duration of menstrual flow, and presence of dysmenorrhea or passage of clots. Values are expressed as number and percentage.

Variable	Category	Number (n)	Percentage (%)
Age at menarche (years)	≤12	20	14.1
	13–14	113	79.6
	≥15	9	6.3
Menstrual cycle	Regular	116	82.0
	Irregular	26	18.0
Duration of flow	3–4 days	58	40.8
	4–5 days	61	43.0
	≥5 days	23	16.2
Dysmenorrhea	Present	12	8.5
Passage of clots	Present	7	4.9

Sanitary pads were used by 95 (66.9%) participants, while 47 (33.1%) used cloth. Knowledge regarding menstrual cup use was present in 87 (61.3%) women and absent in 55 (38.7%) women. Despite this, willingness to use a menstrual cup was high, with 122 (85.9%) expressing willingness and only 20 (14.1%) unwilling (Table 4).

**Table 4:** Menstrual hygiene practices and awareness (n = 142) Type of menstrual hygiene product used, knowledge about menstrual cup, and willingness to use a menstrual cup among participants. Results are presented as number and percentage.

Variable	Category	Number (n)	Percentage (%)
Hygiene product used	Sanitary pad	95	66.9
	Cloth	47	33.1
Knowledge of menstrual cups	Yes	87	61.3
	No	55	38.7
Willingness to use a cup	Yes	122	85.9
	No	20	14.1

At one month, 122 (85.9%) participants continued menstrual cup use and 20 (14.1%) discontinued. At three months, continued use declined to 100 (70.1%), with discontinuation increasing to 42 (29.9%). By six months, 94 (66.2%) women were still using the menstrual cup, while 48 (33.8%) had discontinued, demonstrating a gradual rise in discontinuation over time. All participants were successfully contacted at each follow-up, and discontinuation reflected active cessation of menstrual cup use rather than loss to follow-up. The discontinuation figures at three and six months represent cumulative active refusals (Table 5).

**Table 5:** Dropout trend during follow-up due to refusal of menstrual cup (n = 142) Follow-up status of menstrual cup utilization at one, three, and six months showing continued use versus discontinuation. Percentages are calculated out of the total enrolled participants at each time point.

Utilization status	After one month n (%)	After three months n (%)	After six months n (%)
Continued use	122 (85.9)	100 (70.1)	94 (66.2)
Discontinued	20 (14.1)	42 (29.9)	48 (33.8)
Total	142 (100)	142 (100)	142 (100)

Symptom and utility analyses at each follow-up were calculated among participants who continued menstrual cup use at that specific time point. Thus, denominators differed across time points (n = 122 at one month; n = 100 at three months; n = 94 at six months). Among continuing users, reported symptoms showed a statistically significant declining trend over time. Pain was reported by 109 (89.3%) at one month, decreasing to 21 (21.0%) at three months and to 0 (0.0%) at six months (p < 0.001). Lower abdominal pain was present in nine women (7.4%) at one month and was not reported at three and six months (p < 0.001). Vaginal discharge declined from 30 (24.6%) at one month to 3 (3.0%) at three months and to 0 (0.0%) at six months (p < 0.001). Vaginal irritation reduced from 46 (37.7%) to 4 (4.0%) and then to 0 (0.0%) (p < 0.001). Discomfort decreased from 71 (58.2%) at one month to 12 (12.0%) at three months and to none at six months (p < 0.001). Leakage also declined from 75 (61.5%) to 7 (7.0%) and then to 0 (0.0%) at six months (p < 0.001). At six months, none of the continuing users reported symptoms on the binary self-report assessment (Table 6).

**Table 6:** Symptom-wise feedback regarding menstrual cup use during follow-up Frequency of self-reported symptoms among continuing menstrual cup users at one month (n = 122), three months (n = 100), and six months (n = 94). The chi-square test was used to assess the trend across follow-up periods.

Symptom	Response	One month (n = 122)	Three months (n = 100)	Six months (n = 94)	Chi-square (χ²)	p-value
Pain	Yes	109 (89.3%)	21 (21.0%)	0 (0.0%)	199.5	<0.001
Lower abdominal pain	Yes	9 (7.4%)	0 (0.0%)	0 (0.0%)	14.9	<0.001
Vaginal discharge	Yes	30 (24.6%)	3 (3.0%)	0 (0.0%)	55.8	<0.001
Vaginal irritation	Yes	46 (37.7%)	4 (4.0%)	0 (0.0%)	82.6	<0.001
Discomfort	Yes	71 (58.2%)	12 (12.0%)	0 (0.0%)	118.4	<0.001
Leakage	Yes	75 (61.5%)	7 (7.0%)	0 (0.0%)	138.7	<0.001

Utility parameters showed progressive improvement with continued menstrual cup use. Easy wearing was reported by 108 (88.5%) at one month, increasing to 90 (90.0%) at three months and to 94 (100%) at six months (p = 0.003). Easy removal improved from 114 (93.4%) at one month to 92 (92.0%) at three months and to 94 (100%) at six months (p = 0.025). Ease of cleaning increased markedly from 39 (32.0%) at one month to 76 (76.0%) at three months and to 94 (100%) at six months (p < 0.001). Minor percentage fluctuations between one and three months reflect denominator changes due to discontinuation rather than a reversal of adaptation trends (Table 7).

**Table 7:** Feedback regarding utility of the menstrual cup during follow-up Participant-reported utility parameters (ease of wearing, removal, and cleaning) among continuing users at each follow-up visit. Statistical significance across time points was assessed using the chi-square test.

Utility parameter	Response	One month (n = 122)	Three months (n = 100)	Six months (n = 94)	Chi-square (χ²)	p-value
Easy wearing	Yes	108 (88.5%)	90 (90.0%)	94 (100%)	11.16	0.003
Easy removal	Yes	114 (93.4%)	92 (92.0%)	94 (100%)	8.7	0.025
Easy cleaning	Yes	39 (32.0%)	76 (76.0%)	94 (100%)	106.9	<0.001

## Discussion

The present prospective observational study included 142 married women of reproductive age, with most participants belonging to the 26-35-year age group, specifically 26-30 years (52 (36.6%)) and 31-35 years (48 (33.8%)), representing the prime reproductive period. This age distribution is comparable to the findings of Twisha et al. [18], who reported the majority of participants in the 21-30-year age group [51%], and Garg et al. [5], whose study population ranged between 20 and 36 years. The predominance of participants from upper middle socioeconomic status (83 (58.5%)) and upper class (51 (35.9%)) in the present study is consistent with the findings of Sudevan et al. [19], where most participants belonged to the Above Poverty Line category (293 (83.7%)). This socioeconomic profile may influence access to information, healthcare services, and willingness to adopt newer menstrual hygiene practices.

Regarding obstetric and menstrual characteristics, the study population predominantly consisted of multiparous women, with parity two (40 (28.2%)) and parity three (43 (30.3%)) being most common, while a history of previous LSCS was reported by only 40 women (28.2%). Menarche most frequently occurred at 13-14 years (113 (79.6%)), and the majority of participants reported regular menstrual cycles (116 (82.0%)) with a normal duration of flow of 3-5 days (119 (83.8%)). The prevalence of dysmenorrhea was low (12 (8.5%)), as was the passage of clots (7 (4.9%)), indicating minimal baseline menstrual morbidity. These findings suggest that most participants had physiologically normal menstrual patterns, which is comparable to observations reported in previous studies by Garg et al. and Twisha et al. [5,18].

In the assessment of menstrual hygiene practices, sanitary pads were the most commonly used product (95 (66.9%)), although a considerable proportion continued to use cloth (47 (33.1%)). Similar patterns were observed in studies by Twisha et al. [18], where sanitary napkins were used by 164 (82.5%) participants, and Garg et al. [5], who reported pad usage in 21 (70%) and cloth usage in nine (30%) participants. Awareness regarding menstrual cup use in the present study was moderate (87 (61.3%)), which closely aligns with findings by Garg et al. (18 (60%)) and Bindal et al. (157 (64.2%)) [5,20]. Despite moderate awareness, willingness to use menstrual cups was notably high (122 (85.9%)), consistent with Garg et al. [5], who reported willingness in 26 (86.7%) participants, suggesting that appropriate counselling may significantly enhance acceptance.

Follow-up analyses in the present study were performed among participants who continued menstrual cup use at each time point (n = 122 at one month, n = 100 at three months, and n = 94 at six months), ensuring denominator-corrected interpretation of outcomes. Continued use declined gradually from 122 (85.9%) at one month to 100 (70.4%) at three months and 94 (66.2%) at six months. However, nearly two-thirds of the original cohort sustained use at six months, indicating durable acceptability after initial adoption. It is important to note that discontinuation figures were cumulative and reflected active cessation rather than attrition.

Symptom reporting showed a marked and statistically significant reduction over time among continuing users. Pain decreased from 109 (89.3%) at one month to 21 (21.0%) at three months and to 0 (0.0%) at six months. Discomfort reduced from 71 (58.2%) to 12 (12.0%) and then to 0 (0.0%). Vaginal irritation declined from 46 (37.7%) to 4 (4.0%) and to 0 (0.0%), while leakage reduced from 75 (61.5%) to 7 (7.0%) and subsequently to 0 (0.0%), with all trends being statistically significant (p < 0.001). Similar adaptation patterns, with higher early-cycle complaints that diminish with continued use, have been reported in prior menstrual cup studies [21,22].

However, the absence of reported symptoms at six months among continuing users should be interpreted cautiously. As symptom analysis was restricted to participants who persisted with menstrual cup use, the observed reduction may partly reflect survivorship bias, wherein women experiencing persistent discomfort discontinued earlier. Additionally, symptom assessment relied on binary self-report without a validated severity scale, which may have limited sensitivity for detecting mild symptoms and introduced potential social desirability bias.

Utility parameters demonstrated a progressive learning curve with continued use. Easy wearing increased from 108 (88.5%) at one month to 90 (90.0%) at three months and to 94 (100%) at six months. Easy removal improved from 114 (93.4%) to 92 (92.0%) and to 94 (100%). Ease of cleaning showed the most marked rise, from 39 (32.0%) at one month to 76 (76.0%) at three months and 94 (100%) at six months (p < 0.001). Comparable improvements in comfort and handling with repeated cycles have been described by Kakani et al. and Sreedevi et al. [21,22]. The minor percentage fluctuations observed between one and three months for ease parameters likely reflect denominator changes due to discontinuation rather than a true decline in usability. These findings reinforce that initial handling concerns are typically transient and improve with repeated use and proper guidance.

Mechanistically, early symptoms such as pain, leakage, and irritation may be attributable to improper placement, unfamiliarity with insertion technique, and pelvic floor apprehension during initial cycles. With increasing anatomical familiarity and confidence, these challenges generally diminish. Nevertheless, approximately one-third of participants discontinued use by six months, suggesting that individual anatomical differences, cultural perceptions regarding intravaginal products, or personal preference may limit universal adoption.

Strengths and limitations

The strengths of this study include its prospective observational design, which enabled systematic assessment of menstrual cup use over time, an adequate sample size of 142 participants allowing meaningful evaluation of acceptability and continuation patterns, and multiple follow-up points that captured both early challenges and longer-term adaptation. The consistent statistical significance observed across symptom and utility parameters strengthens the reliability of the findings.

However, certain limitations should be acknowledged. Data were collected from health camps and institutional settings, which may introduce selection bias and limit generalizability. A major methodological limitation is survivorship bias, as symptom and utility analyses were restricted to continuing users at each follow-up; therefore, improvements over time may partially reflect selective retention of satisfied participants rather than true universal symptom resolution. The absence of a comparison group using other menstrual hygiene products restricts direct comparative conclusions, and reliance on self-reported binary responses may be subject to recall bias, reporting bias, and limited measurement sensitivity. Furthermore, the observational single-arm design does not permit definitive conclusions regarding comparative effectiveness or long-term safety.

## Conclusions

The present study shows that menstrual cups are a generally acceptable and practically adoptable menstrual hygiene option for married women of reproductive age, with encouraging continuation and overall user satisfaction over time. Most early-use difficulties and minor symptoms reduced with continued use among continuing users, while comfort and ease of handling improved across follow-up, reflecting a learning and adaptation pattern. No serious adverse events were reported during the study period; however, given the single-arm observational design, definitive conclusions regarding long-term safety cannot be established.

However, as approximately one-third of participants discontinued use by six months, menstrual cups may not be suitable for everyone, and individualized counselling and continued support are essential. Overall, menstrual cups appear to be a sustainable alternative to conventional menstrual products with potential environmental and cost advantages. Further large-scale, controlled, and comparative studies are required to better evaluate long-term safety, effectiveness, and predictors of continued use.

## Appendices

Case record proforma

Case No:

Name:

Age:

Address:

Phone No:

Occupation:

Socioeconomic Status:

Marital history:

Obstetric history:

History of LSCS:  

Menstrual history

Age of menarche:

Cycles: regular/ irregular

Duration

of flow:

Dysmenorrhea:

Clots:

Menstrual hygiene product used:

Knowledge of menstrual cup

Willingness to use cup

**Table 8:** Utilization status

Utilization status	After one month	After three months	After six months
Continued use			
Discontinued			

**Table 9:** Symptom-wise feedback regarding menstrual cup use

Symptom	One month	3 months	6 months
Pain			
Lower abdominal pain			
Vaginal discharge			
Vaginal irritation			
Discomfort			
Leakage			

**Table 10:** Feedback regarding utility of a menstrual cup

Utility parameter	One month	Three months	Six months
Easy wearing			
Easy removal			
Easy cleaning			
